# *doublesex* Functions Early and Late in Gustatory Sense Organ Development

**DOI:** 10.1371/journal.pone.0051489

**Published:** 2012-12-11

**Authors:** David J. Mellert, Carmen C. Robinett, Bruce S. Baker

**Affiliations:** 1 Janelia Farm Research Campus, Howard Hughes Medical Institute, Ashburn, Virginia, United States of America; 2 Biology Department, Stanford University, Stanford, California, United States of America; National Institutes of Health (NIH), United States of America

## Abstract

Somatic sexual dimorphisms outside of the nervous system in *Drosophila melanogaster* are largely controlled by the male- and female-specific Doublesex transcription factors (DSX^M^ and DSX^F^, respectively). The DSX proteins must act at the right times and places in development to regulate the diverse array of genes that sculpt male and female characteristics across a variety of tissues. To explore how cellular and developmental contexts integrate with *doublesex* (*dsx*) gene function, we focused on the sexually dimorphic number of gustatory sense organs (GSOs) in the foreleg. We show that DSX^M^ and DSX^F^ promote and repress GSO formation, respectively, and that their relative contribution to this dimorphism varies along the proximodistal axis of the foreleg. Our results suggest that the DSX proteins impact specification of the gustatory sensory organ precursors (SOPs). DSX^F^ then acts later in the foreleg to regulate gustatory receptor neuron axon guidance. These results suggest that the foreleg provides a unique opportunity for examining the context-dependent functions of DSX.

## Introduction

Studies in *Drosophila melanogaster* have revealed that many complex biological processes, such as the specification of somatic structures [1], [2], segmental identity [3], and sexual differentiation [4], [5], are under the control of master regulatory genes. These genes may act at multiple times in the course of these processes to regulate the expression of distinct sets of target genes. *dsx* is a unique master regulatory gene in that it functions across a wide variety of tissues to specify nearly all aspects of sexual development and differentiation. To manifest its various functions, *dsx* is necessarily responsive to three fundamental contexts: 1) the chromosomal sex of the cells in which it is expressed; 2) cell type; and 3) developmental stage of the cells in which it is expressed.

**Figure 1:**
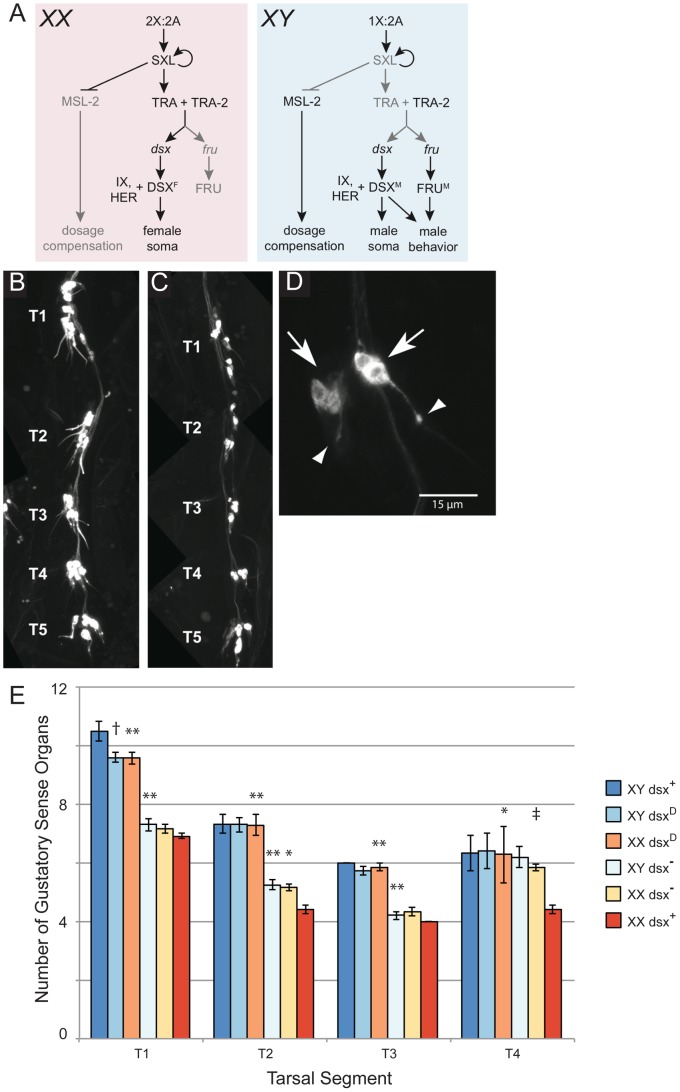
*dsx* regulates the number of foreleg GSOs. (A) The sex determination hierarchy directs the generation of sex-specific DSX and FRU isoforms. The 2∶2 ratio of *X* chromosomes to autosomes in females sets off a female-specific alternative RNA splicing cascade in which TRA directs splicing of *dsx* and *fru* transcripts into the female forms. The lack of TRA activity in males results in the production of male forms of these transcripts. (B–D) *poxn-GAL4* driving expression of *UAS-mCD8::GFP* in a (B) male and (C) female foreleg at 48 h APF. Tarsal segments T1–T5 are indicated. Note that there are more clusters of neurons labeled in the male than in the female in T1–T4. (D) Magnified view of two distinct GSOs. The GRNs (arrows) of each GSO project their dendrites into the base of their cognate bristle (arrowheads). (E) Quantitation of foreleg GSOs in T1–T4. *3XP3DsRed* was used to distinguish *XY* flies from *XX* flies in a *dsx*-deficient background where chromosomal sex could not otherwise be distinguished. All *XY* males had a sex chromosome genotype of *w*/*Y*. The genotype of the sex chromosomes of *dsx-*deficient chromosomal females was *w/w, 3XP3DsRed,* while all other females were *w*/*y w, 3XP3DsRed.* Genotype abbreviations: *dsx* ^+^ (*UAS-mCD8::GFP*; *FRT82B dsx^1^, poxn-GAL4/TM6B*). *dsx* ^−^ (*UAS-mCD8::GFP*; *FRT82B dsx^1^, poxn-GAL4/dsx^M+R13^*). *dsx^D^* (*UAS-mCD8::GFP*; *FRT82B dsx^1^, poxn-GAL4/dsx^D^*). *dsx* ^+^ and *dsx^D^* are siblings from the same cross. Error bars indicate SEM. P-values are for comparisons between the indicated *dsx* mutant and *dsx* ^+^ of the same chromosomal sex. (*p = .07, **p<.0001, † p = .04, ‡ p = .14, Tukey multiple comparisons of means.).

Chromosomal sex impacts *dsx* function via the sex determination hierarchy, which determines whether *dsx* transcripts are spliced to encode the transcription factors DSX^M^ in males or DSX^F^ in females [6] (Fig. 1A). DSX^M^ and DSX^F^ contain a shared zinc finger DNA-binding domain but differ in having sex-specific C-termini [7], [8], [9]. They are thought to bind to, and sex-specifically regulate the transcription of, a common set of target genes. For example, both DSX^M^ and DSX^F^ bind to the enhancer region of the genes encoding Yolk Proteins (YP) 1 and 2, but DSX^M^ down-regulates YP production while DSX^F^ up-regulates YP production [8], [10] **.** Thus, the chromosomal sex of a cell determines the sex-specific functions of *dsx* via post-transcriptional mechanisms.

Transcriptional activation of *dsx* is also under complex spatial regulation [11], [12], such that only specific cells express *dsx* to produce sexually dimorphic traits. These traits include the genitalia, the male-specific sex combs, abdominal segment number and pigmentation, YP production, pheromone production, and gonadogenesis (reviewed in [6], [13], [14]) [15], [16]. *dsx* also controls aspects of neurogenesis [17], [18], [19], [20] and sexual behavior, where it works in conjunction with male-specific functions of *fruitless* (*fru*) to direct sex-specific aspects of nervous system differentiation (reviewed in [21], [22], [23], [24], [25], [26]). Thus, *dsx* regulates a diverse array of developmental programs.

*dsx* also functions in various temporal contexts to regulate developmental programs at the appropriate stage. *dsx* functions in the somatic gonad from embryogenesis through adulthood, while it regulates transcription of the YP genes in the fat body only during adulthood (reviewed in [13], [27]). *dsx* can also function at multiple times within the developmental program of a tissue. In the genital imaginal disc, *dsx* begins functioning early to direct sex-specific patterns of cell proliferation (reviewed in [6], [14]) and then later controls morphogenesis and differentiation of the disc to form sex-appropriate genitalia and analia (reviewed in [6]) [28]. Similarly during foreleg development, *dsx* functions initially to specify the number of bristles in the male-specific sex comb, then subsequently specifies the morphology of these bristles [29]. Thus temporal context, like cell type, constrains the function of *dsx* such that it regulates the right genes at the right times in development.

To understand how *dsx* sculpts sexually dimorphic development, many studies have focused on identifying the genes that *dsx* directly or indirectly regulates (reviewed in [6], [13]) [28], [30], [31], [32], [33], [34], [35], [36], [37], [38], [39], [40], [41]. These studies have offered insight as to how *dsx* regulates diverse developmental processes. First, *dsx* regulates a number of patterning and signaling pathway genes in a sex-specific manner during the development of particular tissues, while these same genes are also expressed independent of *dsx* during the development of other tissues. Second, different genes are regulated by *dsx* in different cell types. A major focus will then be to determine how *dsx* function is integrated with spatiotemporal information such that specific target genes are sex-specifically regulated in appropriate cell types and at appropriate times in development.

Here, we address the function of *dsx* in the development of the gustatory sense organs (GSOs) of the foreleg. GSOs are a class of bristle sensory organs (sensilla) in which multiple gustatory receptor neurons (GRNs) project their dendrites into the shaft of a bristle to detect tastants [42], [43]. The foreleg GSOs of *Drosophila melanogaster* are associated with two sexual dimorphisms. First, males have a greater number of GSOs on their forelegs than do females [43], [44], and second, the axons of many of the constituent GRNs project across the midline of the ventral nerve cord (VNC) in males but not in females [45], [46]. *dsx* has a known role in both of these dimorphisms: it regulates the development of at least some of the GSOs in the first tarsal segment (T1) of the foreleg [44], and represses VNC midline crossing by GRN axons [46].

Our study addresses two questions regarding the role of *dsx* in GSO development. First, we asked if *dsx* is responsible for the sexually dimorphic number of GSOs across all tarsal segments of the foreleg and when this dimorphism is established. Second, we sought to determine if GSO number and GRN axonal projections arise interdependently as the result of a single upstream developmental decision commanded by *dsx* or if these dimorphisms each result from independent developmental events that are individually controlled by *dsx*. We found that *dsx* does control sex-specific GSO numbers early in GSO development and that this function is temporally separable from the previously described role of *dsx* in regulating GRN axonal projections in the VNC. Thus, *dsx* regulates two distinct developmental events that impact the GSO at different times in development.

## Materials and Methods

### Drosophila Stocks

Unless otherwise indicated, crosses were at 25°C under standard conditions. To examine GRNs in *dsx* mutants and controls, *w*; *UAS-mCD8::GFP*; *FRT82B dsx^1^, poxn-GAL4-14-1-7/TM6B* females were crossed to *w, 3XP3-DsRed; dsx^M+R13^/TM6B* males. To generate *dsx*-masculinized females, *y w, 3XP3-DsRed; dsx^D^/TM6B* males were crossed to *w; UAS-mCD8::GFP; FRT82B dsx^1^, poxn-GAL4-14-1-7/TM6B* females. *poxn-GAL4-14-1-7* was from M. Noll [47]. Male parents carrying the *X*-chromosomal transgene *3XP3-DsRed* (from O. Schuldiner [48]) were used to distinguish *XY* (non-fluorescent eyes) and *XX* (fluorescent eyes) *dsx^1^/dsx^M^* ^+*R13*^ and *dsx^1^/dsx^D^* progeny, as these genotypes are otherwise indistinguishable. *neur-lacZ* (*A101*) and *ase-lacZ* were from the Bloomington Drosophila Stock Center at Indiana University (IN, USA).

For post-mitotic expression of *UAS-DSX^F^* [from K. Burtis (University of California, Davis, CA, USA)] in FRU^M^-positive neurons, *w; UAS-mCD8::GFP; fru^GAL4^/MKRS* flies were crossed *to w; UAS-DSX^F^/SM6*. *fru^GAL4^* [49] was from B. Dickson.

### Generation of Anti-DSX^DBD^

The DSX^DBD^ antigen was the generous gift of J. Marvin and L. Looger (Janelia Farm Research Campus, Ashburn, VA, USA). A 60-amino acid polypeptide (MSISPRTPPNCARCRNHGLKITLKGHKRYCKFRYCTCEKCRLTADRQRVMALQTALRRAQ) corresponding to the DNA binding domain (DBD) of the DSX proteins [50] was encoded by a synthetically generated DNA (DNA2.0) and expressed in *E. coli* BL21(pLysS) as a direct fusion to an N-terminal 6-histidine affinity tag in a modified pRSET A vector (Invitrogen). The fusion protein was affinity purified over a Ni^2+^-NTA resin (Qiagen) by an imidazole gradient, and conjugated to keyhole limpet hemocyanin. Hybridoma clones from immunized mice were screened by *in vitro* immunoassay against the DSX^DBD^ polypeptide (RayBiotech, Inc.), and one produced anti-DSX^DBD^, which stains nuclei of third instar larval tissues in patterns consistent with the expression pattern of *dsx^GAL4^* (Fig. S1 and C. Robinett unpublished data) [11]. Anti-DSX^DBD^ does not stain foreleg discs of *dsx* nulls (Fig. S2).

### Preparation and Examination of Tissues

Tissues were fixed in 4% paraformaldehyde (Electron Microscopy Sciences) in phosphate-buffered saline (PBS). Unless otherwise noted, tissues for immunofluorescence were blocked in 5% normal goat serum in PBS containing 0.1% Triton X-100 (Sigma) (PBST), and Alexa Fluor fluorescently conjugated goat secondary antibodies (Molecular Probes/Invitrogen) were used at 1∶500. Tissues were mounted in Vectashield (Vector Laboratories) with or without DAPI. Sample imaging was performed on Zeiss laser scanning confocal microscopes LSM 510 or 710 (Carl Zeiss), and Z-stacks were manipulated using ImageJ (NIH). Images were cropped, rotated, and adjusted for contrast and brightness using Adobe Photoshop.

To examine *poxn-GAL4-*driven expression of *UAS-mCD8::GFP* in 48 h APF forelegs, pupae were removed from puparia in PBS, fixed for 30–45 minutes at 22°C, washed three times in PBS, and examined for native GFP fluorescence. For 2–8 h APF, white pre-pupae were collected at the stage of puparium formation, individually sexed based on the larger size of the male gonads, aged appropriately at 25°C, kept on ice or at 4°C until dissection in PBST, fixed for 25 minutes at 22°C, and washed three times for 15 minutes in PBST. Pupal cuticle secretion around 8 h APF blocks antibody penetration [51], [52], [53], [54], so 8 h APF forelegs were dissected off of fixed pre-pupae by either cutting between T1 and the femur or plucking the entire foreleg away from the pre-pupal carcass. T1 was often damaged or had excessive background staining, preventing assessment of GSO numbers. Dissected legs were blocked in PBST plus 5% normal goat serum (PBSTN) for at least 16 hours at 4°C, incubated in primary mouse monoclonal antibody 22C10 [Developmental Studies Hybridoma Bank at the University of Iowa (DSHB), USA] at 1∶20 for 40–48 hours at 4°C, washed four times in PBST over the course of 10 hours at 22°C or 24 hours at 4°C, and re-blocked in PBSTN overnight at 4°C. Treatment with the secondary antibody, Alexa Fluor 568 anti-mouse was the same as per the primary antibody. Legs were mounted in a circle of nail polish painted onto the slide to prevent flattening of the tissue.

GSO lineage cells were identified based on strong fluorescent signals from both native GFP and 22C10. Inspection of the confocal Z-stack slices determined whether a given cell or cell cluster met our criteria to be a GSO: in T3, a cluster of tightly grouped cells or a large single cell or two large paired cells expressing GFP and stained with 22C10 were classified as GSOs; in T2 and T4, criteria were the same as for T3, but the epithelial expression of *poxn-GAL4*-driven *UAS-mCD8::GFP* required close inspection of morphology, size, and clustering of the cells, which were discounted if they did not appear similar to the GSOs identified in T3.

Larval tissues were immunostained as described in [11]. The DSX^M^ and AC time-course used rat anti-DSX^M^ 5528 (from B. Oliver [55]) at 1∶200 and mouse monoclonal anti-AC (DSHB) at 1∶40 followed by Alexa Fluors 568 anti-rat and 488 anti-mouse. In each time-point preparation, male third instar gonads were included as internal controls; successful immunostaining of gonadal somatic cells confirmed reagent competence when DSX^M^ was not detected in foreleg discs (C. Robinett, unpublished data). For each time-point, a foreleg disc pair (identified by the unique physical association of the two discs) from each of three larvae was mounted in a circle of nail polish (described above). DSX and *ase-lacZ* or *neur-lacZ* expression were detected with anti-DSX^DBD^ at 1∶100 and rabbit anti-β-galactosidase (Cappel/MP Biomedicals, LLC) (1∶500), respectively, followed by Alexa Fluors 488 anti-mouse and 568 anti-rabbit.

For examination of GRN axon morphology, VNCs were dissected from 1-day-old adults and fixed and stained as per [56] using rabbit anti-GFP (Invitrogen) at 1∶1000 and rat anti-DN-cadherin (DN-EX#8) (DSHB) at 1∶40 followed by Alexa Fluors 488 anti-rabbit and 647 anti-rat.

### DSX^M^ and AC Time-course

Canton S larvae were raised at low-density in standard molasses food bottles for ca. 72 hours at 25°C. The food surface was then overlaid with 20% sucrose in H_2_O at 22°C and gently agitated to “float” the larvae into the liquid. 10–30 large larvae having second instar anterior spiracle morphology [57] were transferred to a 2.5-cm diameter food vial supplemented with Brewer’s yeast paste; multiple such vials were set up over the course of an hour before being transferred to 25°C. After 2 hours, larvae were collected from these vials by the method above. Males having third instar anterior spiracle morphology were designated 0 h 3I and returned to 25°C on fresh food with Brewer’s yeast paste for the duration of the time point, whereupon larvae were collected again and held on ice until dissection. Because of the time taken to handle, sort and stage the larvae, all time points are approximations of ±3 hours.

## Results

### *dsx* Specifies the Sexually Dimorphic Number of Foreleg GSOs

Male forelegs have more GSOs than do female forelegs across tarsal segments 1 through 4 (T1–T4) [43], [58], [59]. In T1 (the most proximal tarsal segment), this sexual dimorphism is regulated by *dsx* [44] (wherein GSOs are referred to as “bractless bristles”). We revisited the T1 dimorphism and additionally asked if *dsx* regulates the number of GSOs in T2–T4 by examining the forelegs of males and females that are null for *dsx* function (*dsx^1^*/*dsx^M+R13^*) and comparing them to those with one wild-type copy of *dsx* (*dsx^1^*/*TM6B*, control). GSO cells were marked by expression of *Pox neuro-GAL4-14* (hereafter, *poxn-GAL4*) driving *UAS-mCD8::GFP* [47] and visualized at 48 hours after puparium formation (h APF) as clusters of GRNs whose dendrites converged toward the surface of the leg (Fig. 1B–D). Consistent with previous quantitations of GSOs based on bristle morphology [43], [58], [59], our counts of GSOs on control (*dsx^+^*) male and female forelegs showed that males have more GSOs than do females in tarsal segments T1–T4 (Fig. 1E). In contrast, *dsx* null flies exhibited no significant differences between males and females in the numbers of GSOs in foreleg T1–T4 (Fig. 1E), indicating that sex-specific *dsx* functions are necessary for this sexual dimorphism. Thus, our results with a transheterozygous allelic combination recapitulated the findings of previous experiments with homozygous *dsx^1^* mutants in T1 [44], and further, we found that *dsx* regulates GSO numbers across T2–T4.

To address whether expression of DSX^M^ in *XX* chromosomal females that lacked DSX^F^ is sufficient to produce male-like numbers of GSOs, we examined *dsx^D^*/*dsx^1^* flies in which only male-specific *dsx* transcripts are produced regardless of chromosomal sex (see Materials and Methods) [4], [60]. The number of GSOs in *XX*; *dsx^D^*/*dsx^1^* individuals did not significantly differ from that of their *XY*; *dsx^D^*/*dsx^1^* siblings in T1–T4 (Fig. 1E). Thus, in the absence of DSX^F^, DSX^M^ is sufficient to generate the male number of foreleg GSOs.

The greater number of GSOs in males relative to females could be due to a GSO-promoting action of DSX^M^ in males, a GSO-suppressing action of DSX^F^ in females, or a combination of these two possibilities. When we compared control males and females to *dsx* null flies, we found evidence for each of these three possibilities amongst the foreleg tarsal segments (Fig. 1E). In T1, *dsx* null males and *dsx* null females had an average number of GSOs (7.3±0.2 SEM and 7.2±0.2, respectively) that did not significantly differ from the number of GSOs found in *dsx^+^* control females (6.9±0.1), but were significantly less than the number of GSOs present in dsx^+^ males (10.5±0.3). Similarly in T3, *dsx* null males and *dsx* null females had an average number of GSOs (4.2±0.1 and 4.3±0.1, respectively) that did not significantly differ from the number of GSOs in *dsx^+^* control females (4.0±0.0), but were significantly less than the number of GSOs present in dsx^+^ males (6.0±0.0). Thus in T1 and T3, DSX^M^ induces the male-specific number of GSOs, whereas DSX^F^ appears to have no effect on the number of GSOs. In T4, the number of GSOs in both *dsx* null males and *dsx* null females (6.2±0. and 5.8±0.1, respectively) were roughly equal to the number present in *dsx^+^* control males (6.3±0.6) and higher than the number in *dsx^+^* control females (4.4±0.1). These data indicate that in T4, DSX^F^ represses the development of two GSOs, while DSX^M^ has no effect. In T2, *dsx* null males and females had numbers of GSOs (5.3±0.2 and 5.2±0.1, respectively) that differed significantly from both control males (7.3±0.3) and females (4.4±0.1). Thus in T2, DSX^M^ promotes GSO formation in males, while DSX^F^ represses GSO formation in females.

We conclude that the wild-type function of *dsx* ensures that each of the T1–T4 tarsal segments elaborates a greater number of GSOs in males than in females, but that this shared outcome arises by differential use of DSX^M^ and DSX^F^ in the tarsal segments. This distinction implies that positional information is integrated with sexual identity (i.e. *dsx* expression) on a fine-scale along the proximodistal axis of the developing foreleg.

#### The sexually dimorphic number of GSOs is established by 8 h APF

**Figure 2:**
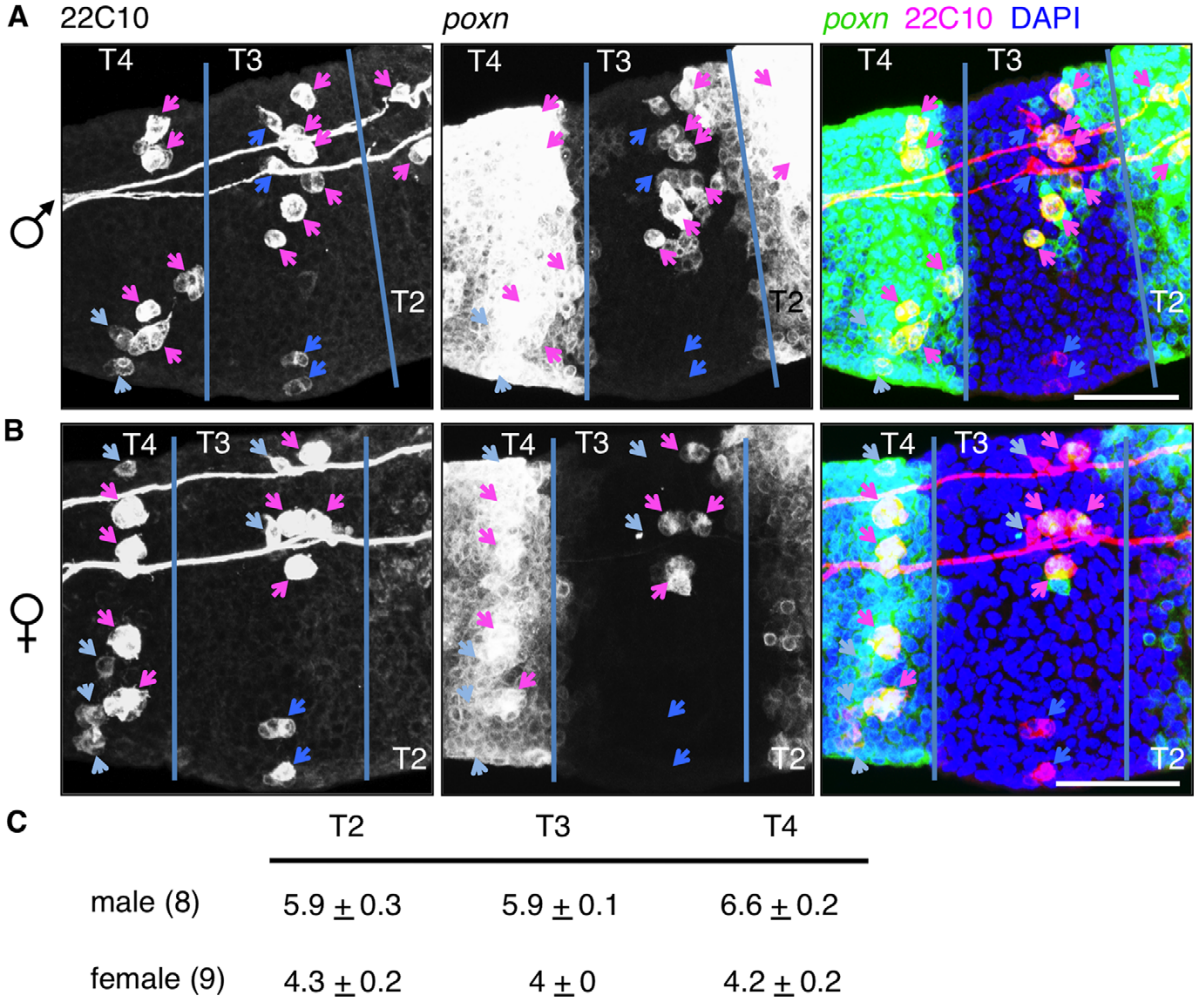
The sexually dimorphic number of foreleg GSOs is specified by 8 h APF. Male (A) and female (B) forelegs with *poxn-GAL4* driving *UAS-mCD8::GFP* (green) were stained for 22C10 (magenta) at 8 h APF. Merged images on right show overlap in yellow and DAPI-stained DNA (blue). Tarsal segment boundaries indicated with blue lines. Cells marked with 22C10 were classified based both on colocalization of *poxn-GAL4* and morphology of the cells or cell clusters: GSO lineage cells (magenta arrows); non-GSO cells that lack *poxn-GAL4* in T3 (dark blue arrows); non-GSO cells marked by *poxn-GAL4* but lacking GSO morphology in T4 (light blue arrows). Scale bars, 50 µm. (C) Averages and SEMs of quantitated GSO numbers in T2, T3, and T4 for both male (n = 8) and female (n = 9) forelegs at 8 h APF.

To address how *dsx* function specifies the sex-specific number of foreleg GSOs, we sought to determine when this sexual dimorphism was first apparent. We found that GSOs were identifiable by morphology and cell clustering as early as 14 h APF using the *poxn-GAL4* marker and that the sexual dimorphism in GSO number was already apparent (C. Robinett, unpublished data). Examination of earlier time points was complicated by the expression of *poxn-GAL4* across the leg disc epithelium of T2 and T4 from 0–6 h APF (Figs. 2 and S3), regions where *poxn* is required for formation of the intertarsal joints [61]. Because cells of the GSOs could not be clearly distinguished from epithelial cells using *poxn-GAL4* alone, we incorporated staining with the 22C10 monoclonal antibody, which marks both neurons and other cell types of nascent sensory organs [62] and thus allows us to positively identify the GSOs.

In 8 h APF foreleg discs, GSOs could be recognized as multi-cell clusters that exhibited strong signals for both GFP and 22C10 (see Materials and Methods) (Figs. 2A,B and S4). There were also single large cells and pairs of large cells exhibiting both GFP and 22C10 signals at strengths similar to what was observed for cells within the GSO cell clusters (Figs. 2A,B, S4 and S5). Given that 22C10 marks nascent sensory organ cells, we consider these large cells to be the single sensory organ precursor cells (SOPs), which each give rise to a single GSO, or to the first pair of daughters of an SOP, respectively. As both of these cell types represent the GSO lineage, they were equated to GSOs in our analysis. We counted GSOs in male and female tarsal segments T2–T4 and found that the numbers were the same as seen in adults (Fig. 2C, compare to Fig. 1E). (T1 was not examined, see Materials and Methods). Therefore, the sexual dimorphism in foreleg GSOs number is established prior to 8 h APF. Because a subset of gustatory SOPs appeared to be undergoing their first cell division around 8 h APF, the developmental mechanism that establishes the sexually dimorphic number of GSOs likely acts prior to the division of gustatory SOPs.

#### DSX is present in proneural clusters and SOPs

It has been reported that the foreleg gustatory SOPs are specified before or near the time of puparium formation [52]. We therefore examined the distribution of DSX protein in T2–T4 of the foreleg disc before and at 0 h APF using anti-DSX^DBD^, a monoclonal antibody that recognizes both DSX^M^ and DSX^F^ (see Materials and Methods and Figs. S1 and S2). At this stage of development, foreleg discs of male and female larvae exhibited DSX proteins in a crescent of epithelial cells that occupied the T1 domain as well as in smaller patches of epithelial cells in T2–T4 (Fig. S2A). DSX was not detected across the T5 disc epithelium or in regions of the foreleg disc that were proximal to T1, and there was no staining of the disc epithelium in other leg discs at this stage (Fig. S2D,F,G). This distribution of DSX is in accord with the expression pattern of the *dsx* promoter [11]. Although the distribution of DSX appeared to be the same in male and female foreleg discs, males showed stronger signal intensity (Fig. S2A,B).

We note two important features in the distribution of DSX. First, the number of epithelial cells marked by DSX^M^ is far greater than the number of GSOs in the adult foreleg, indicating that *dsx* expression is not restricted to the SOPs that will give rise to the GSOs. Second, the distribution of DSX in broad swaths of epithelial cells is restricted to those leg tissues that produce sexually dimorphic GSO numbers, as foreleg segment T5 and the tarsus of the second leg exhibit sexually monomorphic bristles [43], [59], [63]. These data thus suggest that *dsx* may regulate GSO numbers by exerting its function broadly across the epithelium of tarsal segments T2–T4 at a time preceding SOP specification.

**Figure 3:**
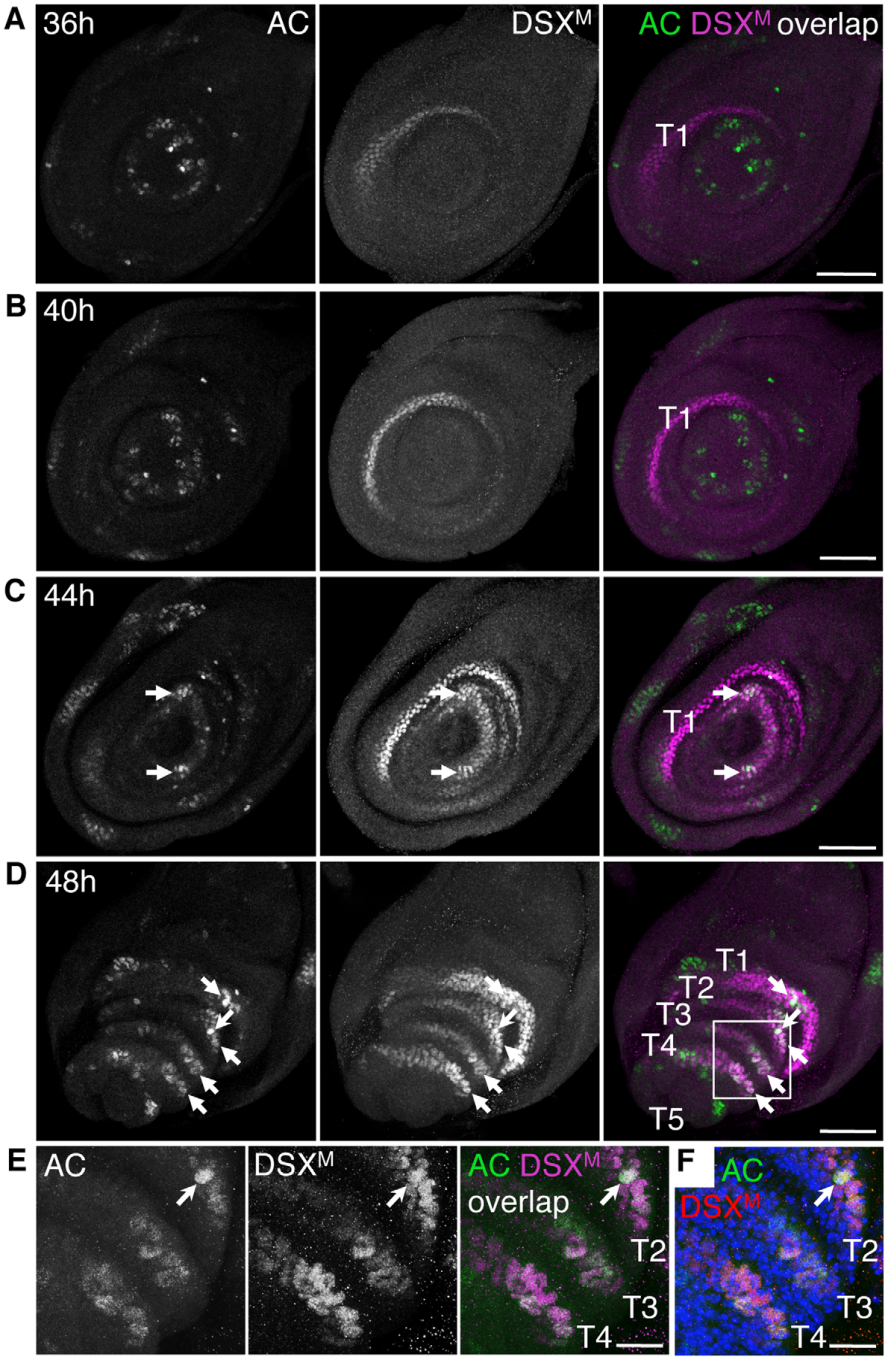
DSX^M^ is present in the foreleg disc epithelium when AC accumulates in proneural clusters. (A–E) Male foreleg discs from the indicated time points of third instar larval development were stained for AC (green) and DSX^M^ (magenta). Merged images on right show overlap in white. (A and B) From 36–40 h 3I, DSX^M^ is present in a crescent within T1 and there is no overlap with AC. (C) At 44 h 3I, DSX^M^ signal increases across the epithelium of tarsal segments distal to T1 (i.e. toward disc center) and is present in some clusters of AC-positive cells (arrows). (D) At 48 h 3I, DSX^M^ is present in swaths of epithelial cells in T1–T4 and overlaps in these segments with subsets of the AC-positive cells that are proneural clusters (arrows). A candidate SOP with high levels of AC and DSX^M^ (barbed arrow) is seen in T2. (E) Magnified view of boxed region in (D). Candidate SOP in T2 (barbed arrow). (F) Same image as (E) with AC (green), DSX^M^ (red), and stained with DAPI (blue) to visualize all nuclei in the focal planes shown. All images are projections of only those focal planes that encompass the majority of DSX^M^ signal within the disc. Scale bars (A–D) 50 µm and (E and F) 10 µm.

In the wing imaginal disc, specification of the thoracic mechanosensory organ SOPs depends on the expression of the proneural genes *achaete* (*ac*) and *scute* (*sc*), which confer neural potential to patches of epithelial cells called proneural clusters from which the SOP cell is selected (reviewed in [64], [65], [66]). To ascertain whether *dsx* might function in proneural clusters of the foreleg disc, we determined the pattern of DSX^M^ with respect to Achaete (AC) in foreleg discs of male late third instar larvae. Nascent third instar larvae (0 h 3I) were isolated, raised at 25°C, and then analyzed at 4-hour increments from 24 h 3I until the time of puparium formation at 48 h 3I, which is equivalent to 0 h APF. At 24 h 3I, AC was detected only in a few cells of T5, while DSX^M^ was absent from the disc (Fig. S7A). AC-positive cells were observed at a greater number in T5 at 28 h 3I (Fig. S7B), as well as in more proximal domains at 32 h 3I (Fig. S7C). However, DSX^M^ was not detected in the foreleg disc until 36 h 3I, when it weakly marked a crescent of epithelial cells in T1 (Fig. 3A). This staining signal intensified at 40 h 3I, and by 44 h 3I DSX^M^ appeared in swaths of epithelial cells in the more distal tarsal segments, T2–T4 (Fig. 3B,C). The pattern of AC also became more complex during 36–44 h 3I as multiple patches of AC-positive cells were distributed across the proximal and distal regions of the foreleg disc (Fig. 3A–C). Importantly, there was significant overlap between DSX^M^ and AC in patches of cells distal to T1 at 44 h 3I, and this pattern became more pronounced in T2–T4 at 48 h 3I (Fig. 3C,D). Because AC marks cells with proneural potential, we consider these co-expressing patches of cells to be nascent proneural clusters. Further, because leg mechanosensory sense organs are not specified until around 8 h APF [51], [52], we assume that these proneural clusters will give rise to gustatory SOPs.

**Figure 4:**
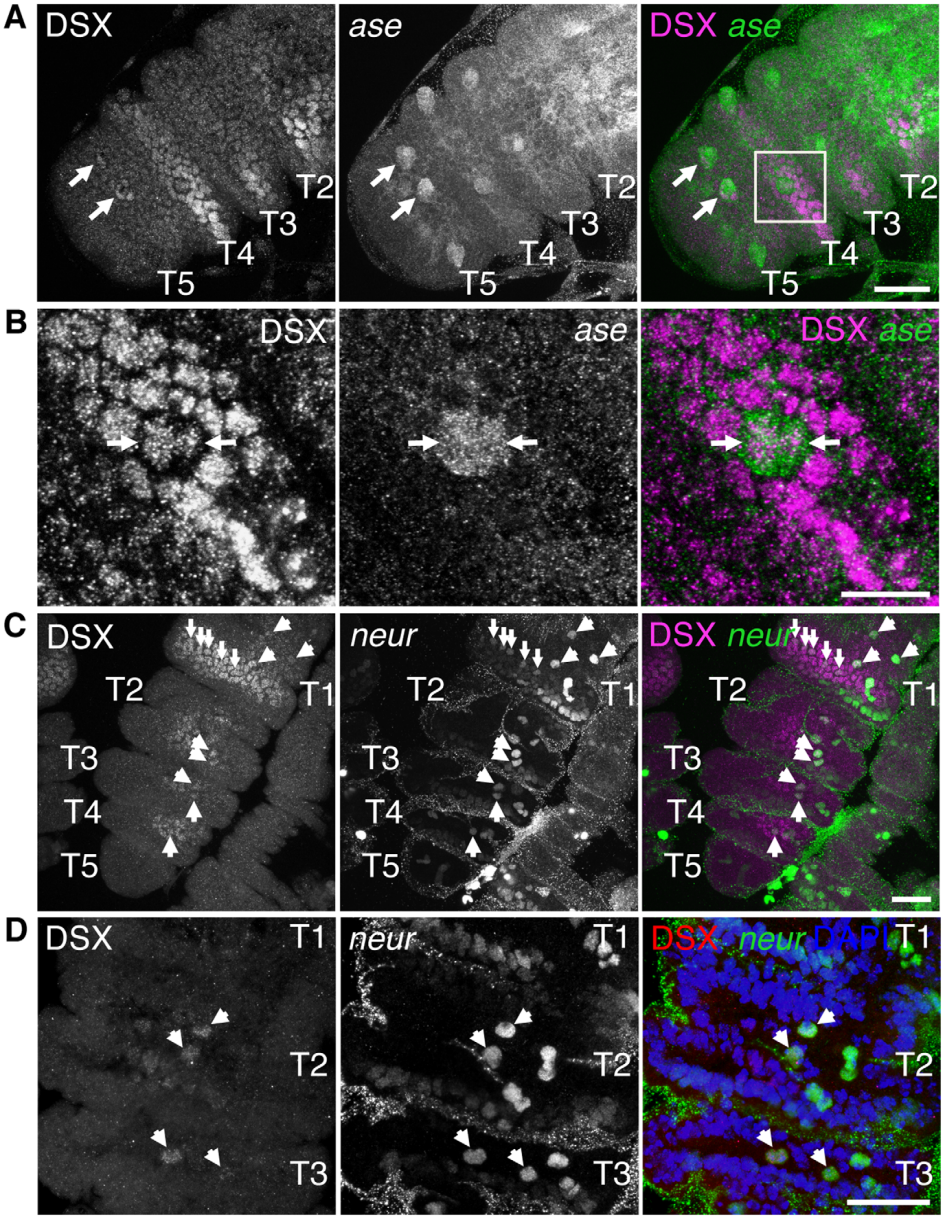
DSX is present in SOP daughters of the foreleg disc. (A and B) DSX (magenta) is present across the tarsal segment epithelium in male discs at 0 h APF as well as in subsets of cells expressing *ase-lacZ* (green) in T5 (arrows) and T4 (boxed area). (B) Magnified view of boxed region in (A) shown as a partial projection. Daughters of a recently divided SOP (arrows). (C) DSX (magenta) is present in the tarsal segment epithelium in male discs at 6 h APF. DSX overlaps with *neur-lacZ* expression (green) in several cells across T1–T5 (arrowheads) and in a transverse row of cells in T1 that likely correspond to the sex comb bristle lineages (small arrows). (D) T2–T3 from a separate male leg disc at 6 h APF marked as per (C) with DSX (red) in right panel and DAPI-stained DNA (blue). For A–D, images on right are a merge of the left and middle images. Projection of multiple focal planes shown. Projection of multiple focal planes shown. Scale bars (A, C, and D) 25 µm and (B) 10 µm.

Once an SOP is specified within the proneural cluster, it accumulates high levels of AC and Scute (SC) and concomitantly initiates expression of the neural precursor gene *asense* (*ase*), which in the embryonic nervous system and wing imaginal disc promotes the SOP fate and ensures proper development of sensory organs [67], [68], [69], [70]. To determine if DSX^M^ is present early in cells of the GSO lineage following SOP specification, we examined the distribution of DSX^M^ with respect to *ase-lacZ*, a cytoplasmic reporter of *ase* expression that is expressed in SOPs and their first daughter cells [68]. In male foreleg discs at 0 h APF, DSX^M^ was present in many of the disc epithelial cells within T1–T4, as well as in the previously mentioned clusters of cells in T5 (Fig. 4, see also Fig. 2). In all tarsal segments, *ase-lacZ* was expressed in distinct clusters of cells that generally resembled the pattern seen with *poxn-GAL4* at later time-points, and some of these cells also contained DSX^M^ (Fig. 4A,B), as indicated by the nuclear-localized DSX^M^ signal surrounded by cytoplasmic β-galactosidase immunoreactivity. In T5, the cells containing DSX^M^ were part of a cluster of cells that expressed *ase-lacZ* (Fig. 4A). In T4, DSX^M^ was seen in a pair of *ase-lacZ* cells whose cytoplasm occupied a larger volume than the surrounding epithelial cells (Fig. 4B). That the *ase-lacZ* cytoplasmic staining corresponded to only two cells was confirmed by nuclear staining (Fig. S6). This pair of tightly associated cells expressing *ase-lacZ* is assumed to be the immediate daughters of a recently divided SOP. Thus, DSX^M^ is present in the immediate progeny of at least a subset of SOPs in the foreleg tarsal segments.

We also examined the distribution of DSX^M^ with respect to *neuralized-lacZ* (*neur-lacZ*), a marker of SOPs and their progeny [71], [72], at the later time-point of 6 h APF. DSX^M^ was present in patches of cells on the anterior surface of T1–T4, with broader expression in cells of distal T1 (Fig. 4C,D). Across T1–T4, DSX^M^ colocalized with the expression of *neur-lacZ* in a few large cells that are likely to be gustatory SOPs (Fig. 4C,D), which is consistent with the presence of dividing *poxn-GAL4*-expressing cells that were positive for 22C10 at 6 h APF (Fig. S8). Whether the presence of DSX^M^ in a subset of gustatory SOPS results from either continued expression of *dsx* in these cells or perdurance of the protein from expression in cells of the proneural clusters, we conclude that *dsx* is expressed at a time and place that would allow it to regulate the specification of the gustatory SOP fate.

### DSX^F^ can Repress Midline Crossing by GRN Axons Independent of its Regulation of Neurogenesis

We previously showed that DSX^F^ in females represses VNC midline crossing by GRN axonal projections [46]. Two possibilities were proposed to account for this phenomenon: 1) DSX^F^ regulates axon guidance in foreleg GRNs to prevent midline crossing in females; or 2) DSX^F^ prevents midline crossing by preventing the birth of male-specific neurons that would send axonal projections across the VNC midline. If DSX^F^ regulates axon guidance independent of its role in regulating GSO formation, then it should be possible to perturb *dsx* function in such a way as to preserve the wild-type number of male-specific GSOs but alter the axonal morphology of their GRNs. Alternatively, if DSX^F^ acts only to regulate GSO numbers, then perturbing *dsx* function in males after GSO number has been specified should not affect GRN axonal morphology.

**Figure 5:**
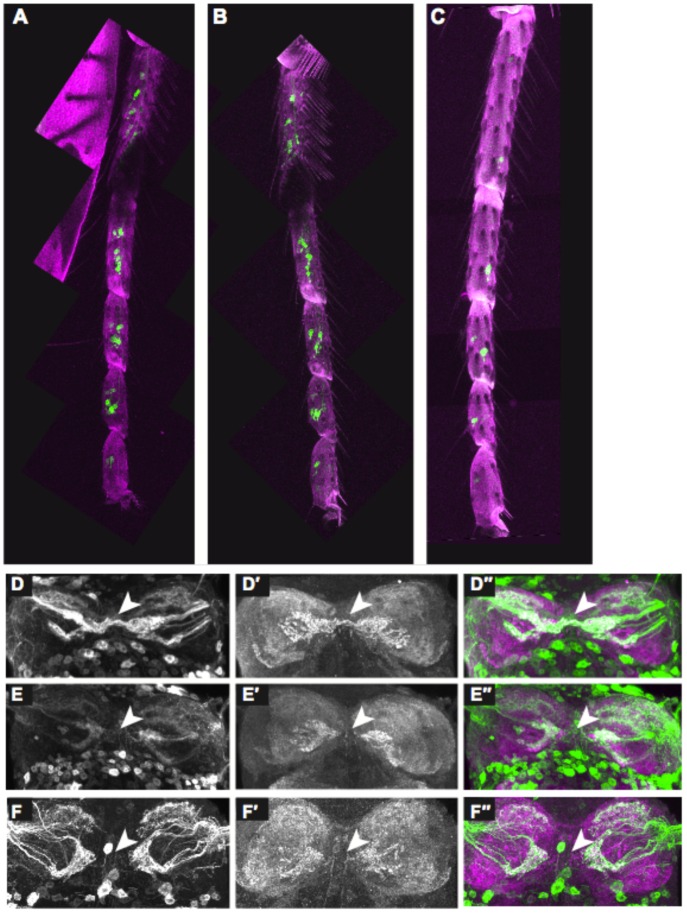
*dsx* regulates axonal morphology independent of GSO number. (A–F”) *fru^GAL4^* driving *UAS-mCD8::GFP* (green) labels GRN cell bodies and axons. Forelegs of (A) control male (*UAS-mCD8::GFP/SM6; fru^GAL4^/+* ), (B) male with feminized GRNs (*UAS-DSX^F^/UAS-mCD8::GFP; fru^GAL4^/+*), and (C) control female (*UAS-mCD8::GFP/SM6; fru^GAL4^/+*). Cuticular autofluorescence (magenta). There is no difference in the number of FRU^M^-positive GRN clusters between forelegs of the two male genotypes, while both have more than the female. (D–F”) VNC prothoracic neuromeres with labeled GRN projections (D, E, F; green in D”, E”, F”) and counterstained for DN-cadherin (D’, E’, F’; magenta in D”, E”, F”). Arrowheads indicate the VNC midline. (D–D”) GRNs cross the midline in control males, but feminized male GRNs do not cross (E–E”). (F–F”) GRNs also do not cross the midline in control females.

We tested these hypotheses by expressing DSX^F^ in nascent male GRNs during axonal development. Specifically, we drove *UAS-DSX^F^* with *fru^GAL4^* [49] which initiates expression in postmitotic GRNs before their nascent axons encounter the VNC midline (D. Mellert, unpublished data). To assess behavior of GRN axons at the VNC midline, *fru^GAL4^* expression was visualized by simultaneously driving *UAS-mCD8::GFP* while the sensory neuropil was highlighted by counterstaining DN-cadherin [73]. Surprisingly, DN-cadherin staining alone was sufficient to assess midline crossing (see Fig. 5D’,E’,F’), and corroborated the findings for *fru^GAL4^*-driven expression of *UAS-mCD8::GFP*. We crossed *w; UAS-mCD8::GFP; fru^GAL4^* females to *w; UAS-DSX^F^/SM6* males and examined three classes of progeny: control males and females that carried the *SM6* chromosome, and males that expressed *UAS-DSX^F^* under the control of *fru^GAL4^*. As expected, control males (Fig. 5A) had more *fru^GAL4^*-expressing GRNs in their forelegs than control females (Fig. 5C), and only in control males were GRNs observed to have crossed the VNC midline (Fig. 5D, F). In contrast, when DSX^F^ was expressed in the *fru^GAL4^*-expressing GRNs, midline crossing was eliminated (Fig. 5E), even though the number of GSOs appeared the same (Fig. 5B). This indicates that the roles of DSX^F^ are temporally separable during development of the female foreleg: DSX^F^ first acts early to regulate neurogenesis then acts in the terminally differentiated neuron to regulate axon guidance. Thus, *dsx* functions in two distinct developmental contexts within the GSO lineage.

## Discussion

We report that *dsx* regulates the sexually dimorphic number of GSOs across all tarsal segments of the foreleg: DSX^M^ promotes and DSX^F^ represses the development of certain GSOs. The effects of this regulation are apparent by 8 h APF, when the GSOs are first identified, and the spatiotemporal pattern of DSX implies that *dsx* determines the number of gustatory SOPs. *dsx* exhibits a surprising degree of context sensitivity: the relative importance of DSX^M^ and DSX^F^ varies along the proximodistal axis of the foreleg and, during the course of GSO development, DSX^F^ progresses from regulating cell fate to regulating axon guidance.

Given that *dsx* controls the formation of the other sexually dimorphic cuticular structures of the fly, as well as the number of GSOs in segment T1 of the foreleg [44], we anticipated that *dsx* would regulate the sex-specific GSO numbers in segments T2–T4 of the foreleg. However, the manner in which this regulation is achieved across the tarsal segments was surprising. Although each of the T1–T4 foreleg tarsal segments produces more GSOs in males than in females, in two segments this difference is achieved by promoting formation of several GSOs in males (via the action of DSX^M^), in one segment it is achieved by repressing the formation of several GSOs in females (via the action of DSX^F^), and in another segment, both DSX^M^ and DSX^F^ act to regulate GSO number. This is more complicated than the simpler *a priori* expectation that the function of *dsx* would be the same across the T1–T4 foreleg segments.

That DSX^M^ and DSX^F^ can be utilized differentially has been previously established. In the fat body, female-specific expression of *Yp1* and *Yp2* depends on up-regulation by DSX^F^ in females and down-regulation by DSX^M^ in males [8], [10], [74]. Thus, in *dsx* null flies, both sexes express these genes at equivalent levels. Similarly, DSX^F^ activates and DSX^M^ represses expression at the *bric-a-brac* locus to generate sex-specific pigmentation in the abdominal epithelium [75]. In these two cases, both DSX proteins contribute to regulation of a single trait, similar to the regulation of GSO number in T2. In contrast, *desatF* is activated by DSX^F^ in oenocytes to produce female-specific pheromones without influence from DSX^M^ [31]. This single isoform-mediated regulation bears similarity to the regulation of foreleg GSOs in T1, T3 and T4. Whereas the previous studies found that DSX^M^ or DSX^F^ were differentially utilized to sculpt sexually dimorphic traits arising from developmentally distinct tissues, we have found that these transcription factors can be differentially utilized across a single developmental field–the epithelium of the foreleg disc. Moreover, the differential roles of DSX^M^ and DSX^F^ in different tarsal segments suggest that each segment may have independently evolved a molecular mechanism for integrating sexual and proximodistal axis information within the foreleg disc to produce more GSOs in the male.

We also sought to determine when the function of *dsx* impacts neurogenesis to generate the numbers of GSOs. Although the details of foreleg GSO development have not been specifically reported, studies of the mechanosensory macrochaete lineages of the notum provide a basic framework for the multi-step process of sensory organ neurogenesis (reviewed in [64], [65], [76], [77]). The initiating event is patterned expression of the proneural genes *ac* and *sc*, which imparts the potential to produce SOPs to specific clusters of epithelial cells across the disc epithelium. Subsequent cell-cell interactions within the cluster typically specify a single SOP (see also [78], [79], [80]). The nascent SOP must then sustain its fate and undergo a series of stereotyped cell divisions to produce all of the cells of the sensory organ (reviewed in [76], [77]). Any of the molecular processes that underlie these stages could be influenced by the functions of *dsx*.

We were struck by the broad distribution of the DSX proteins across the T1–T4 foreleg disc epithelium before and at 0 h APF, a time when the gustatory SOPs are specified [52]. Because the number of DSX-positive cells far exceeded the number of gustatory SOPs necessary to give rise to the GSOs, we infer that *dsx* is acting prior to or during SOP formation. This is consistent with the frequent colocalization of DSX with AC in proneural clusters, which suggests that *dsx* might act within these cells to determine whether the SOP fate is promoted in males or repressed in females. The broad distribution of the DSX proteins could ensure that sexual information is available for integration with positional information across the foreleg disc epithelium to guide sexually dimorphic development.

In contrast to T2–T4, DSX was not apparent in the foreleg epithelium of T5, which produces a sexually monomorphic GSO number. Thus, the presence of DSX in the epithelium correlates with the adult sexual dimorphism in GSO number, consistent with the notion that *dsx* is expressed at the right time and place to impact SOP selection in the foreleg. However, at 0 h APF we observed DSX in two nascent sensory organs expressing *ase-lacZ* (Fig. 4A, and C. Robinett, unpublished data) [67], [68]. We speculate that these sensory organs correspond to the GSOs containing GRNs that express *pickpocket 25* (*ppk25*), which is enriched in males and required for their normal response to female pheromones [81], [82]. Thus, the presence of DSX in the nascent GSOs may forecast sexually dimorphic gene expression in the adult GSO.

In addition to specifying foreleg GSO numbers, we observed a temporally distinct function for *dsx* in the GRNs. During pupal development, GRN axons project proximally along the leg nerves and into the VNC, and here the behavior of the axon depends on the activities of FRU^M^ or DSX^F^. In males, FRU^M^ promotes crossing of the VNC midline by the axons, but in females, DSX^F^ represses this behavior [46]. This dual regulation causes GRN axons to project across the VNC midline only in males [45]. We previously proposed two competing hypotheses to explain the action of DSX^F^: 1) DSX^F^ directly affects axon guidance in differentiating GRNs; or 2) only male-specific GRNs are competent to cross the VNC midline and DSX^F^ indirectly affects midline crossing by repressing formation of the male-specific GSOs. Having shown that post-mitotic expression of DSX^F^ in Fru^M^-expressing GRNs subsequent to the establishment of GSO number prevents midline crossing, we now reject the second hypothesis. Moreover, the early sexual information that impacts GSO number does not irreversibly determine sex-specific development of the GRNs as they continue to be sensitive to the action of DSX^F^ (and presumably FRU^M^). Because *dsx* and *fru* are classically thought of as acting in parallel, we were intrigued to find both genes regulating the same phenotype in a common set of GRNs. Determining whether they coregulate a common set of target genes or independently regulate distinct targets will be of great interest.

Because DSX^M^ and DSX^F^ differentially impact GSO numbers in different tarsal segments, and DSX^F^ regulates the later process of axon guidance, the identity of the genes directly regulated by *dsx* during foreleg development likely changes with spatiotemporal context. Although we currently do not know which genes are directly regulated by *dsx* in the foreleg epithelium or the GSO lineage, the available data on *in vivo* DSX binding sites [41] may reveal genes that are known to be involved in peripheral neurogenesis or axon guidance. The challenge will then be to determine if such candidates exhibit sexually dimorphic expression in the different tarsal segments at different developmental time points. In this way, development of the foreleg GSOs presents a unique opportunity for investigating how *dsx* function is integrated with spatiotemporal context across a changing developmental landscape.

## Supporting Information

Figure S1 **Immunoreactivity of anti-DSX^DBD^.** Late third instar larval tissues stained with anti-DSX^DBD^ (white in A–F; green in A’–F’) and shown as partial projections of a confocal stack. Images A’–F’ are merged with DAPI-stained DNA (blue). (A, A’) Low resolution dorsal view of brain and VNC. (B, B’) Clusters of labeled nuclei (arrows) in posterior of brain. (C, C’) Labeled nuclei in the abdominal ganglion of the VNC. (D, D’) Labeled epithelial cell nuclei in tarsal segments of the foreleg imaginal disc. (E, E’) Labeled fat body nuclei. (F, F’) Labeled somatic cell nuclei of the male gonad include cyst cells (arrows) and the posterior cells (bracket). Scale bars (A,B) 50 µm and (C–F) 25 µm.
(TIF)

Figure S2 **Immunoreactivity of anti-DSX^DBD^ is specific to DSX.** (A–B, E–G) Tissue from mature third instar larvae stained with anti-DSX^DBD^ (white in A–G, red in A’ and B’, green in E’–G’) and merged with DAPI-stained DNA (blue in A’–G’). (A, A’) Wild-type male foreleg disc. (B, B’) Wild-type female foreleg disc, shown in lower magnification than male. (C and D) Wild-type male foreleg disc (C) and second leg disc (D) at 0 h APF showing distribution of immunoreactivity across foreleg tarsal segments. Tibia (tib). Note clusters of DSX-positive cells in T5 (arrows). (E,E’) *dsx* mutant foreleg disc homozygous for the *dsx* deficiency *Df(3R)f00683-d07058*, which was generated by FLP/FRT-mediated deletion of the native chromosomal sequence between piggyBac insertions *f00683* and *d07058,* as per the methods of Parks et al. [*Nat. Gen.* 36(3):288-92. 2004]. Note loss of immunoreactivity. (F, F’) Wild-type male foreleg discs, second leg disc, and partial ventral view of CNS. Note that the second leg disc lacks immunoreactivity. (G, G’) Magnifed view of second leg disc from (F, F’). Scale bars (A, B, E) 50 µm, (C, D, G) 25 µm and (F) 100 µm.
(TIF)

Figure S3 **Expression of** ***poxn-GAL4*** **in the larval leg disc after puparium formation.** 22C10 labels cells of the nascent gustatory sensillum, while *poxn-GAL4* driving *UAS- mCD8::GFP* is expressed across the epithelium of T4 and T2. (A) 0 h APF. (B) 2 h APF. (C) 6 h APF. Each sample is compressed ot a different degree. Scale bars, 25 µm.
(TIF)

Figure S4 **View of whole 8-h APF forelegs shown in** **Fig. 2** **.** Male (A) and female (B). The left panels show 22C10 staining, while the right panels are a merge of 22C10 (magenta), *poxn-GAL4* driving *UAS-mCD8::GFP* (green), and DNA stained with DAPI (blue). Tarsal segments boundaries are indicated with light blue lines in left panels. Cells marked with 22C10 were classified based on both colocalization of *poxn-GAL4* and morphology of the cells or cell clusters: GSO lineage cells (magenta arrows); non-GSO cells that lack *poxn-GAL4* in T1 and T3 (dark blue arrows); non-GSO cells marked by *poxn-GAL4* but lacking GSO morphology in T2 and T4 (light blue arrows). In panel (A), the row of 22C10-positive cells (bracket) in T1 are likely the sex comb SOPs. Scale bars, 50 µm.
(TIF)

Figure S5 **Gustatory SOPs and daughter cells at 8 h APF.** Male foreleg disc at 8 h APF with *poxn-GAL4* driving *UAS-mcd8::GFP* (green), stained with 22C10 (red) and DAPI (blue). (A) Whole foreleg disc. Cells marked with the barbed arrowhead or arrowhead are enlarged in (B) and (C), respectively. (B) A large single cell in T3 is likely to be a pre- mitotic SOP. (C) Pair of large cells in T2 in which the lower cell has metaphase chromosomes (arrow). Note that *poxn-GAL4* is expressed strongly in all cells of the T2 epithelium. Projections of confocal slices shown. Scale bars, (A) 50 µm and (B,C) 10 µm.
(TIF)

Figure S6 **DSX is present in the daughters of a recently divided SOP.** Shown is DAPI staining of the *ase-lacZ*–expressing, anti-DSX^DBD^-stained cells from Fig. 4B. Two masses of DNA can be distinguished (green arrows), indicating separate nuclei. This pair of tightly associated cells expressing *ase-lacZ* is assumed to be the immediate daughters of a recently divided SOP.
(TIF)

Figure S7 **DSX^M^ is not present in the male foreleg disc epithelium at 32 h 3I or preceding time points.** (A–C) Male foreleg discs from the indicated time points of third instar larval development were stained for AC (left panels) and DSX^M^ (middle panels). Right panels show merged images of DSX^M^ (magenta), AC (green) and DAPI-stained DNA (blue). From 24–32 h 3I, DSX^M^ is not detected in the foreleg disc, while AC is present in single cells and cell clusters mostly in T5 at the center of the discs. The number of AC-positive cells increases over time. All images are projections of only those confocal sections that encompass the majority of AC signal from a given disc as no DSX^M^ signal was detected. Scale bars, 50 µm.
(TIF)

Figure S8 **Some gustatory SOPs are dividing at 6 h APF.** (A and B) T4–T2 region of male foreleg disc with *poxn-GAL4* driving *UAS-mCD8::GFP* (green in middle and right panels) stained with DAPI (white in left panel, blue in middle and right panels) and 22C10 (red in right panel). (A) Several 22C10-positive cells expressing *poxn-GAL4* have mitotic figures indicating cell division (arrows). (B) Enlargement of the dividing cell in the T3 region of (A) (arrow). Projection of only a few confocal sections shown. Scale bars (A) 10 µm and (B) 5 µm.
(TIF)
