# Supplementary figures and images for: doublesex Functions Early and Late in Gustatory Sense Organ Development
Source: PLoS One. 2012 Dec 11;7(12):e51489. doi: 10.1371/journal.pone.0051489 (PMC3519885; doi:10.1371/journal.pone.0051489)

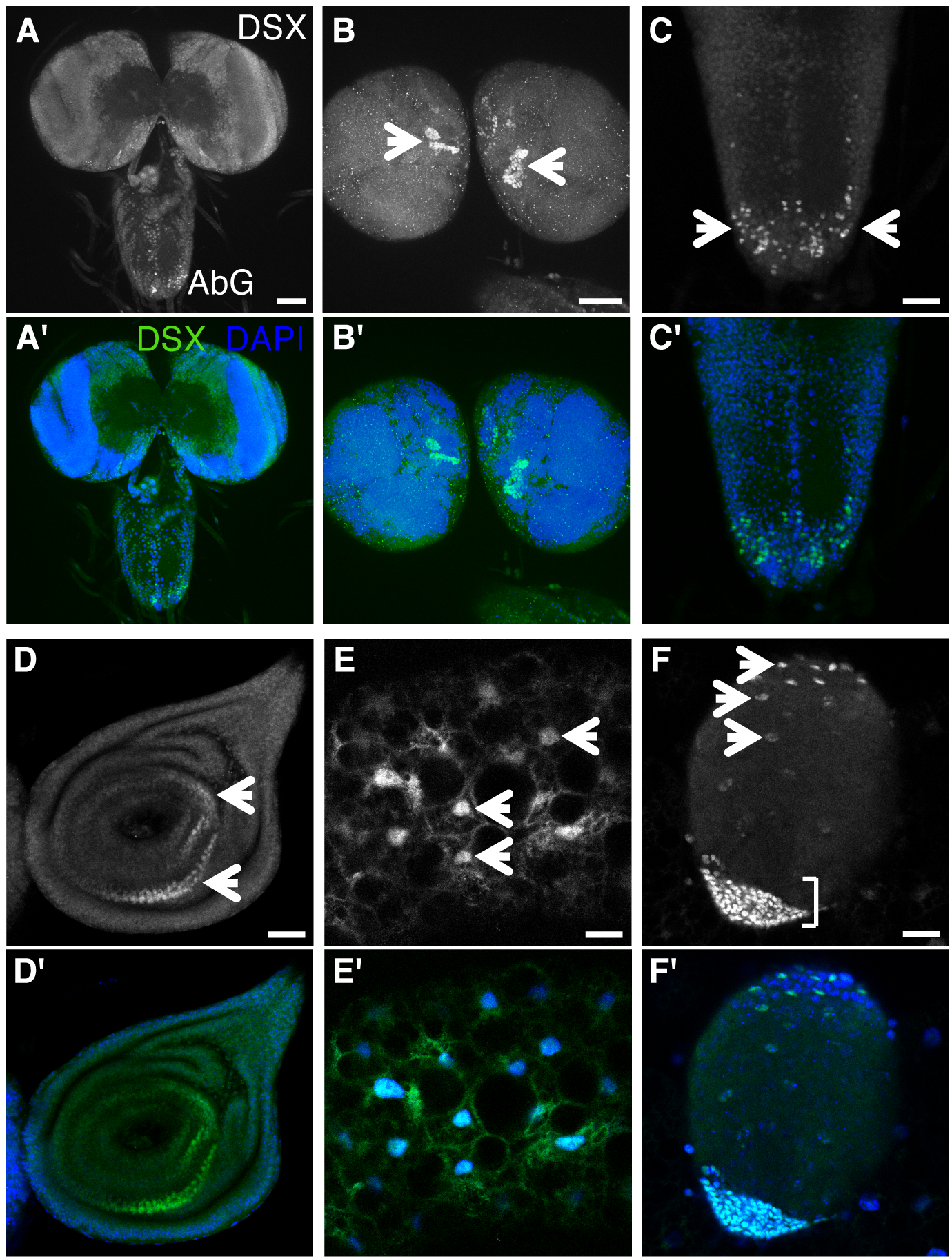

Supplement: Figure S1 — Immunoreactivity of anti-DSXDBD. Late third instar larval tissues stained with anti-DSXDBD (white in A–F; green in A’–F’) and shown as partial projections of a confocal stack. Images A’–F’ are merged with DAPI-stained DNA (blue). (A, A’) Low resolution dorsal view of brain and VNC. (B, B’) Clusters of labeled nuclei (arrows) in posterior of brain. (C, C’) Labeled nuclei in the abdominal ganglion of the VNC. (D, D’) Labeled epithelial cell nuclei in tarsal segments of the foreleg imaginal disc. (E, E’) Labeled fat body nuclei. (F, F’) Labeled somatic cell nuclei of the male gonad include cyst cells (arrows) and the posterior cells (bracket). Scale bars (A,B) 50 µm and (C–F) 25 µm. (TIF) [file pone.0051489.s001.tif]

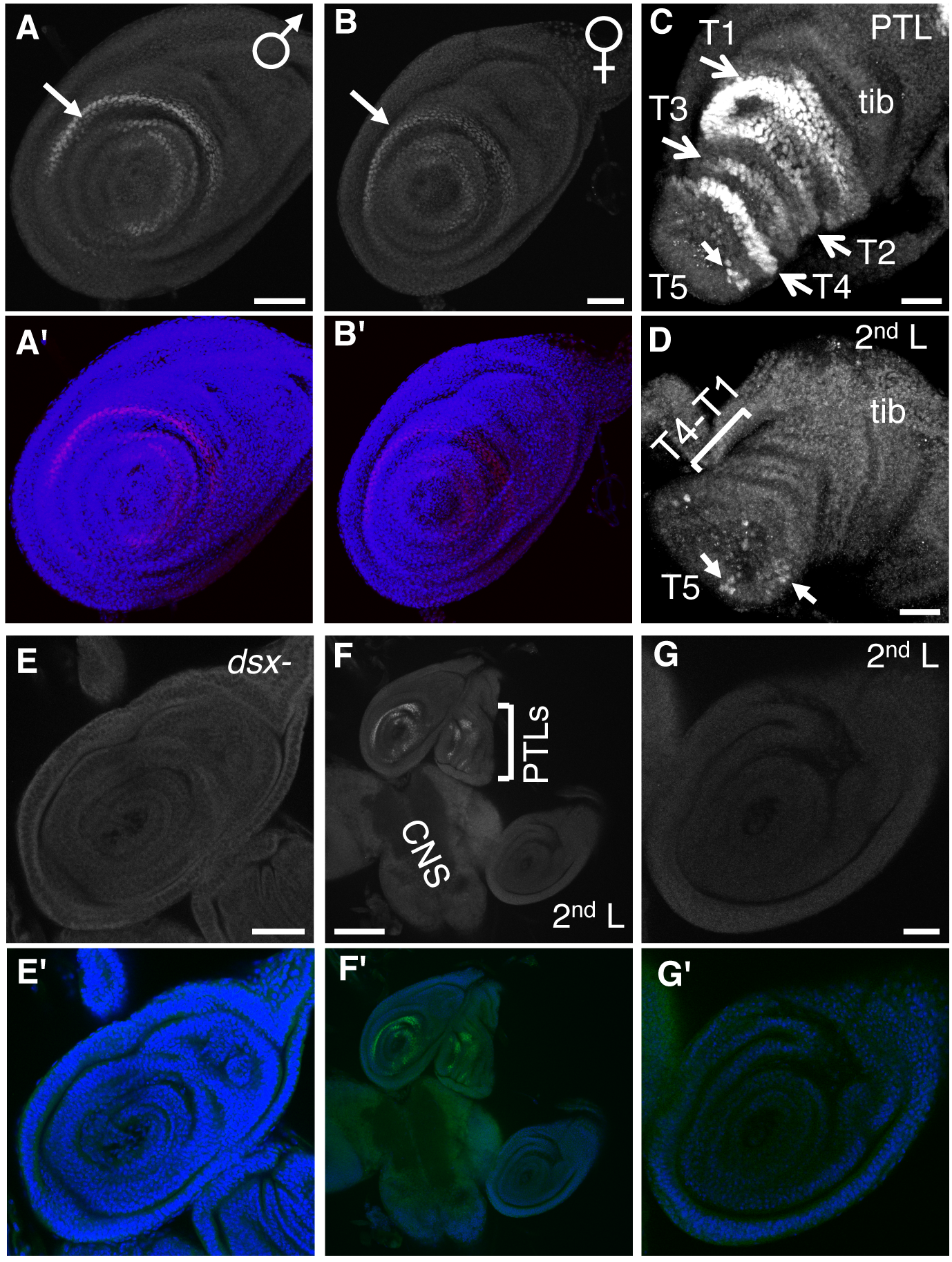

Supplement: Figure S2 — Immunoreactivity of anti-DSXDBD is specific to DSX. (A–B, E–G) Tissue from mature third instar larvae stained with anti-DSXDBD (white in A–G, red in A’ and B’, green in E’–G’) and merged with DAPI-stained DNA (blue in A’–G’). (A, A’) Wild-type male foreleg disc. (B, B’) Wild-type female foreleg disc, shown in lower magnification than male. (C and D) Wild-type male foreleg disc (C) and second leg disc (D) at 0 h APF showing distribution of immunoreactivity across foreleg tarsal segments. Tibia (tib). Note clusters of DSX-positive cells in T5 (arrows). (E,E’) dsx mutant foreleg disc homozygous for the dsx deficiency Df(3R)f00683-d07058, which was generated by FLP/FRT-mediated deletion of the native chromosomal sequence between piggyBac insertions f00683 and d07058, as per the methods of Parks et al. [Nat. Gen. 36(3):288-92. 2004]. Note loss of immunoreactivity. (F, F’) Wild-type male foreleg discs, second leg disc, and partial ventral view of CNS. Note that the second leg disc lacks immunoreactivity. (G, G’) Magnifed view of second leg disc from (F, F’). Scale bars (A, B, E) 50 µm, (C, D, G) 25 µm and (F) 100 µm. (TIF) [file pone.0051489.s002.tif]

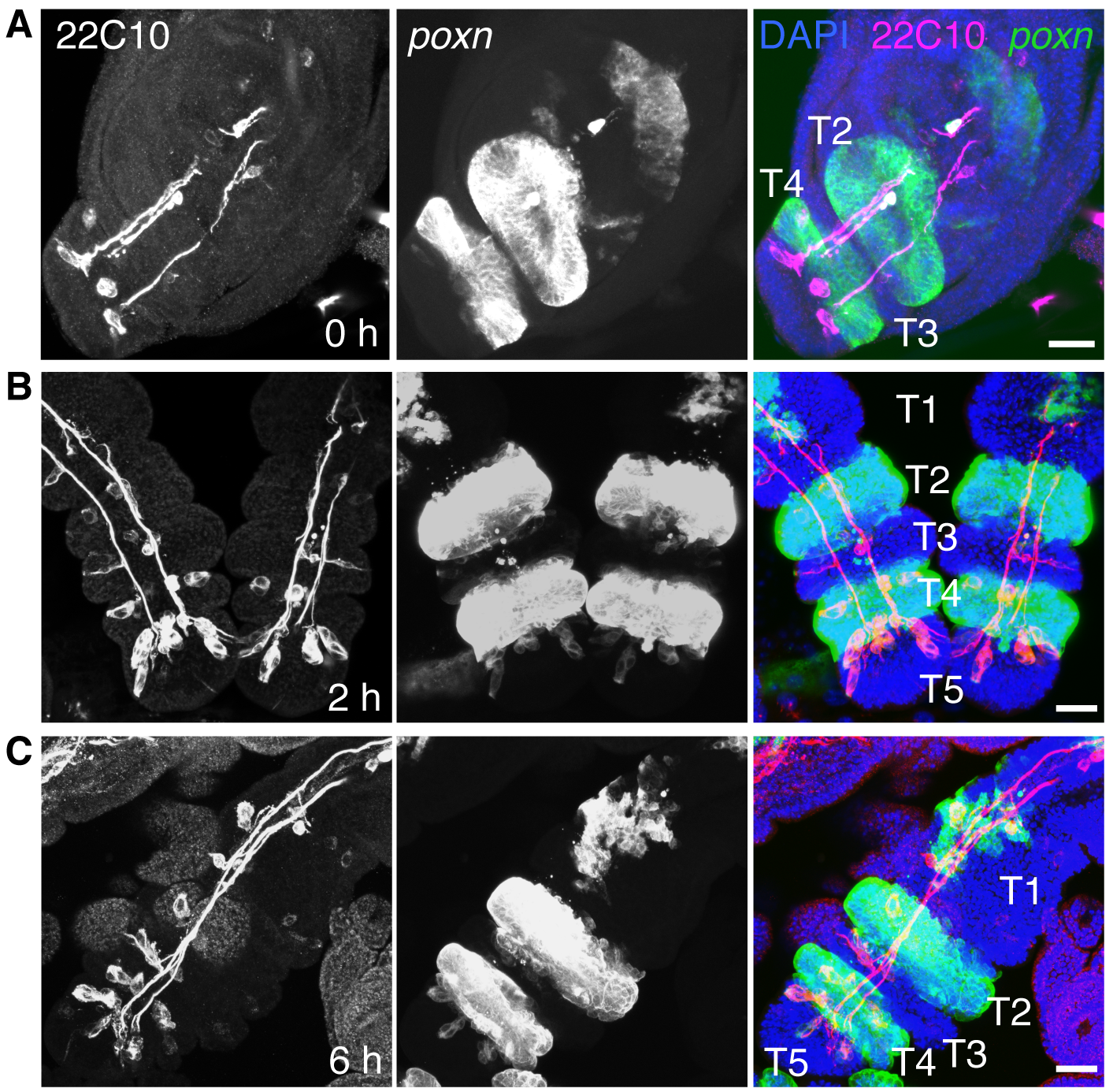

Supplement: Figure S3 — Expression of poxn-GAL4 in the larval leg disc after puparium formation. 22C10 labels cells of the nascent gustatory sensillum, while poxn-GAL4 driving UAS- mCD8::GFP is expressed across the epithelium of T4 and T2. (A) 0 h APF. (B) 2 h APF. (C) 6 h APF. Each sample is compressed ot a different degree. Scale bars, 25 µm. (TIF) [file pone.0051489.s003.tif]

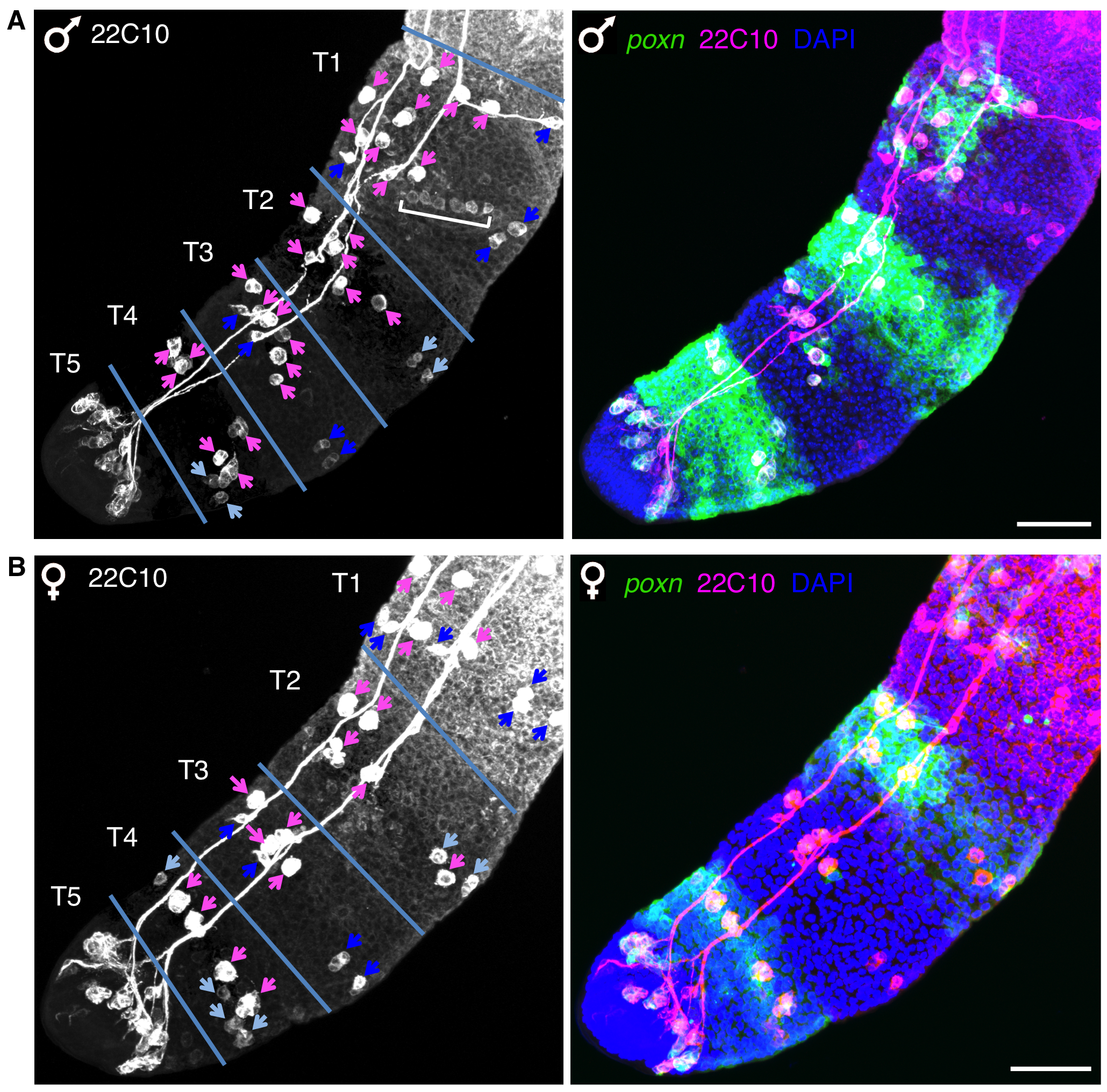

Supplement: Figure S4 — View of whole 8-h APF forelegs shown in Fig. 2 . Male (A) and female (B). The left panels show 22C10 staining, while the right panels are a merge of 22C10 (magenta), poxn-GAL4 driving UAS-mCD8::GFP (green), and DNA stained with DAPI (blue). Tarsal segments boundaries are indicated with light blue lines in left panels. Cells marked with 22C10 were classified based on both colocalization of poxn-GAL4 and morphology of the cells or cell clusters: GSO lineage cells (magenta arrows); non-GSO cells that lack poxn-GAL4 in T1 and T3 (dark blue arrows); non-GSO cells marked by poxn-GAL4 but lacking GSO morphology in T2 and T4 (light blue arrows). In panel (A), the row of 22C10-positive cells (bracket) in T1 are likely the sex comb SOPs. Scale bars, 50 µm. (TIF) [file pone.0051489.s004.tif]

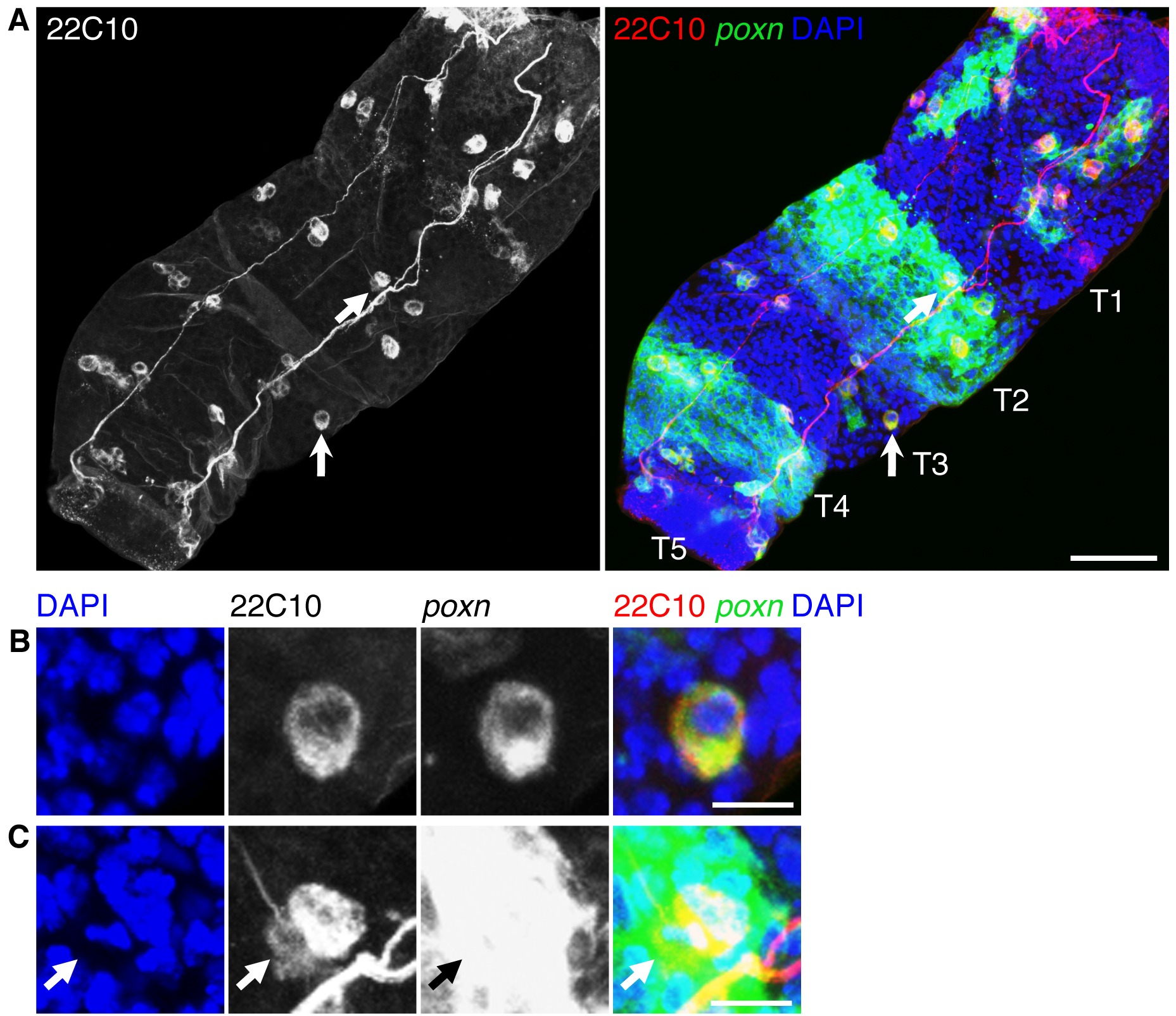

Supplement: Figure S5 — Gustatory SOPs and daughter cells at 8 h APF. Male foreleg disc at 8 h APF with poxn-GAL4 driving UAS-mcd8::GFP (green), stained with 22C10 (red) and DAPI (blue). (A) Whole foreleg disc. Cells marked with the barbed arrowhead or arrowhead are enlarged in (B) and (C), respectively. (B) A large single cell in T3 is likely to be a pre- mitotic SOP. (C) Pair of large cells in T2 in which the lower cell has metaphase chromosomes (arrow). Note that poxn-GAL4 is expressed strongly in all cells of the T2 epithelium. Projections of confocal slices shown. Scale bars, (A) 50 µm and (B,C) 10 µm. (TIF) [file pone.0051489.s005.tif]

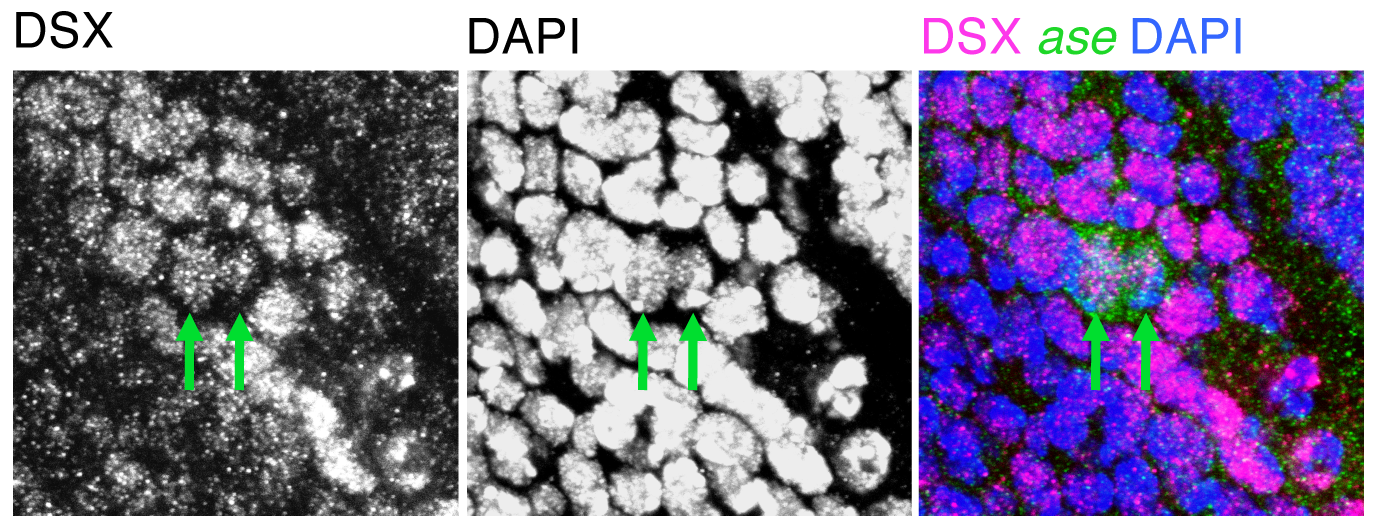

Supplement: Figure S6 — DSX is present in the daughters of a recently divided SOP. Shown is DAPI staining of the ase-lacZ–expressing, anti-DSXDBD-stained cells from Fig. 4B. Two masses of DNA can be distinguished (green arrows), indicating separate nuclei. This pair of tightly associated cells expressing ase-lacZ is assumed to be the immediate daughters of a recently divided SOP. (TIF) [file pone.0051489.s006.tif]

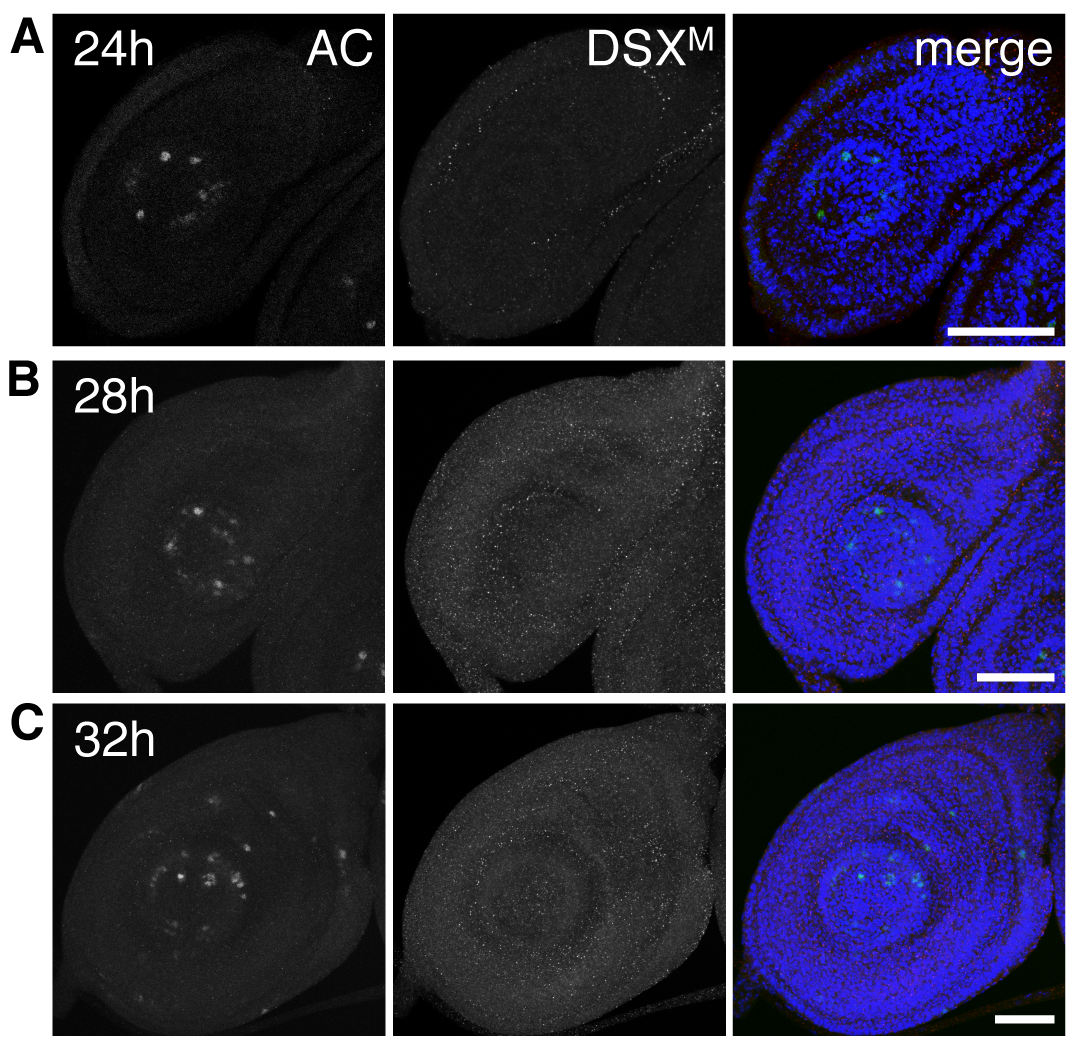

Supplement: Figure S7 — DSXM is not present in the male foreleg disc epithelium at 32 h 3I or preceding time points. (A–C) Male foreleg discs from the indicated time points of third instar larval development were stained for AC (left panels) and DSXM (middle panels). Right panels show merged images of DSXM (magenta), AC (green) and DAPI-stained DNA (blue). From 24–32 h 3I, DSXM is not detected in the foreleg disc, while AC is present in single cells and cell clusters mostly in T5 at the center of the discs. The number of AC-positive cells increases over time. All images are projections of only those confocal sections that encompass the majority of AC signal from a given disc as no DSXM signal was detected. Scale bars, 50 µm. (TIF) [file pone.0051489.s007.tif]

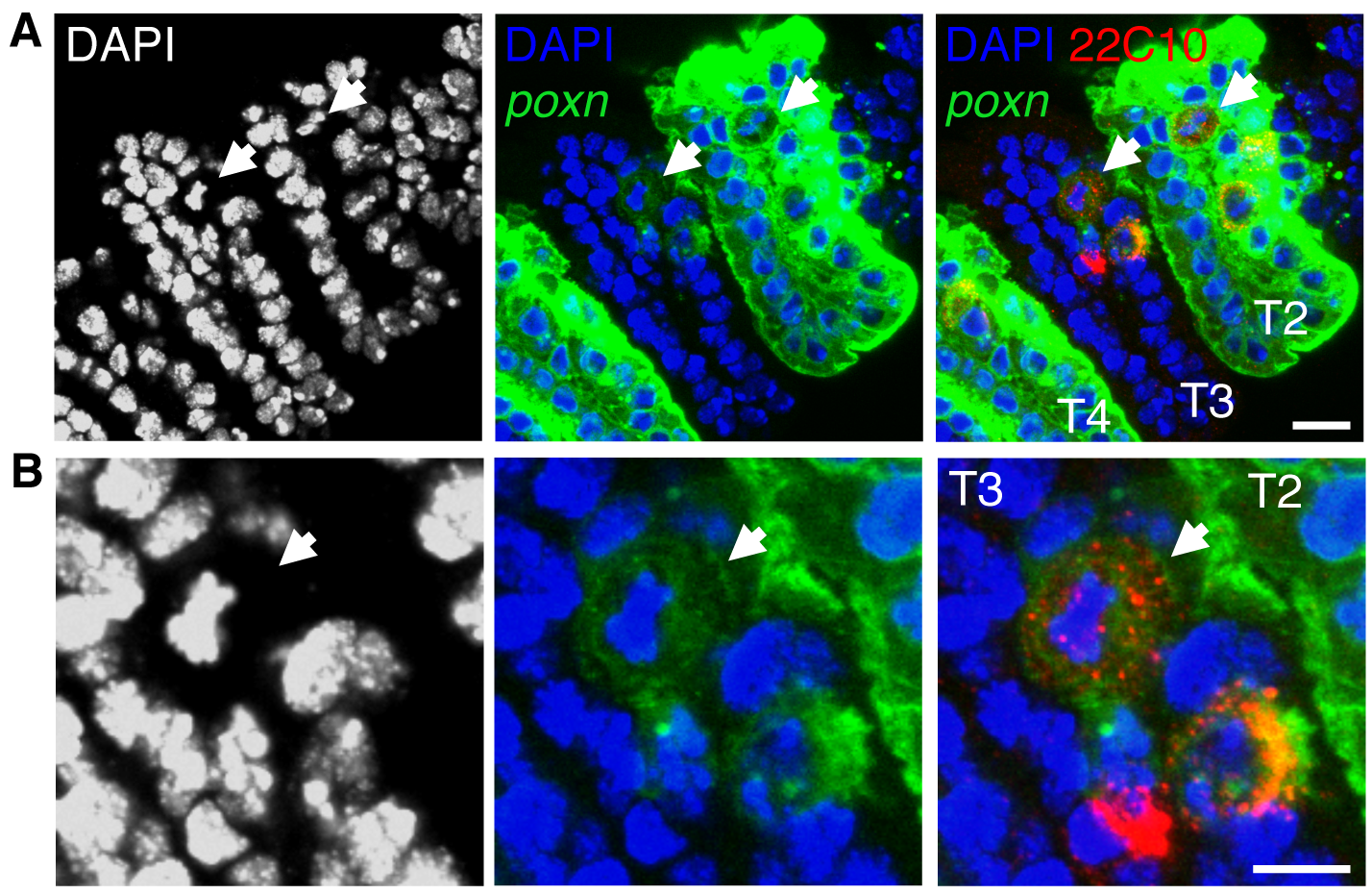

Supplement: Figure S8 — Some gustatory SOPs are dividing at 6 h APF. (A and B) T4–T2 region of male foreleg disc with poxn-GAL4 driving UAS-mCD8::GFP (green in middle and right panels) stained with DAPI (white in left panel, blue in middle and right panels) and 22C10 (red in right panel). (A) Several 22C10-positive cells expressing poxn-GAL4 have mitotic figures indicating cell division (arrows). (B) Enlargement of the dividing cell in the T3 region of (A) (arrow). Projection of only a few confocal sections shown. Scale bars (A) 10 µm and (B) 5 µm. (TIF) [file pone.0051489.s008.tif]
